# Analysis of endometrial histopathological classifications and associated factors in infertile women with heterogeneous endometrial echogenicity

**DOI:** 10.1097/MD.0000000000050528

**Published:** 2026-09-04

**Authors:** Saihua Ma, Rong Dong, Jiao Liu, Jingyu Xu, Baojuan Wang, Linlin Fan, Zheng Gong, Tian Xia

**Affiliations:** a Reproductive Center, First Teaching Hospital of Tianjin University of Traditional Chinese Medicine, Tianjin, China; b Reproductive Center, National Clinical Research Center for Chinese Medicine, Tianjin, China; c Graduate School, Guangzhou University of Chinese Medicine, Guangzhou, Guangdong, China.

**Keywords:** endometrial heterogeneity, hysteroscopy, infertility, pathological diagnosis

## Abstract

Infertility is a key reproductive health challenge, with endometrial lesions as a major cause. Heterogeneous endometrial echo is an initial screening tool, and hysteroscopy plus pathology is the diagnostic gold standard. This study explored pathological subtypes, risk factors of heterogeneous echo in infertile women, and predictive models’ performance. This study analyzes the histopathological classifications and associated influencing factors of endometrial tissue in infertile women with heterogeneous endometrial echogenicity identified on ultrasound. It also seeks to explore the clinical value of such echogenic patterns in diagnosing endometrial lesions, thereby providing evidence-based support for the clinical diagnosis and management of these patients. This was a cross-sectional study that enrolled infertile patients undergoing hysteroscopy due to heterogeneous endometrial echogenicity. We analyzed the histopathological classifications of the endometrium and its associated influencing factors. A total of 312 infertile women with heterogeneous endometrial echo were retrospectively analyzed. Clinical data, hysteroscopic findings, and pathology results were collected. Group comparisons, Spearman correlation, logistic regression, receiver operating characteristic curves/confusion matrices, neural network, and restricted cubic spline were used. A total of 227 (72.8%) had endometrial lesions (chronic endometritis: 175, 56.09%; polyps: 85, 27.24%; hyperplasia: 106; fibroids: 2); 85 (27.24%) were negative. Body mass index, infertility duration, and endometrial thickness correlated positively with lesions (*P* < .05); infertility type correlated negatively (*r*=−0.158, *P* = .005). Body mass index (BMI) and endometrial thickness were independent risk factors. The exploratory neural network analysis identified endometrial thickness as the most important predictor (normalized importance score = 0.301), followed by BMI (0.245) and age (0.229). The internally validated multivariable logistic regression model achieved the area under the receiver operating characteristic curve of 0.701 (0.637–0.760) and the area under the precision-recall curve of 0.859 (0.808–0.902). At the Youden-optimal threshold of 0.733, sensitivity and specificity were 0.652 and 0.670, respectively. Restricted cubic spline analysis suggested a nonlinear association, with the estimated adjusted odds ratios increasing modestly above the 10-mm reference value. Chronic endometritis and polyps are common in these women. BMI, endometrial thickness, and infertility duration are key risk factors. BMI and endometrial thickness were independently associated with endometrial lesions, while the internally validated prediction model showed modest discrimination, emphasizing ultrasound monitoring and weight management for diagnosis/treatment.

## 1. Introduction

According to data from the National Bureau of Statistics of China, the number of births in the country has declined for 7 consecutive years, with a negative population growth reported for the first time in 2022. The continuous rise in infertility rates is considered one of the key contributors to the declining birth rate.^[1]^ The World Health Organization’s report “Infertility Prevalence Estimates, 1990 to 2021” indicated that approximately 17.5% of adults of reproductive age worldwide: roughly 1 in every 6 individuals: are affected by infertility, with the prevalence showing an upward trend. This has made infertility a significant public health issue of global concern.^[2]^

Good endometrial receptivity is a critical factor for successful embryo implantation and the maintenance of pregnancy. Among the various indicators for assessing endometrial receptivity, the endometrial echogenic pattern is considered one of the simplest and most commonly used. Transvaginal sonography (TVS) serves as an essential tool for evaluating endometrial morphology and echogenicity. According to the 2015 consensus statement released by the International Endometrial Tumor Analysis group under the Morphological Uterus Sonographic Assessment guidelines, heterogeneous endometrial echogenicity may present as an endometrium with or without cystic structures or as a uniformly echogenic endometrium with accompanying cystic changes.^[3]^

Clinical ultrasound studies have shown that heterogeneous endometrial echo may represent physiological variants in some women, especially during hormonal cycling, but is often associated with pathological changes such as endometrial hyperplasia (EH), endometrial polyps (EP), chronic endometritis (CE), and malignancy. A recent prospective study based on International Endometrial Tumor Analysis guidelines confirmed that heterogeneous echogenicity, along with increased endometrial thickness and atypical vascular patterns, are significant predictors of endometrial malignancy, including EH and EP.^[4]^ Another review emphasized that EPs are frequently associated with abnormal bleeding and may appear as heterogenous structures on ultrasound, especially in women aged 40 to 49.^[5]^ These findings highlight the diagnostic value of heterogenous endometrial echo patterns in identifying both benign and malignant lesions. These pathological conditions are closely associated with infertility, miscarriage, and repeated implantation failure.^[6,7]^

TVS is widely used in clinical practice due to its noninvasive nature, safety, and ease of operation, making it an important screening tool for endometrial lesions. However, in cases where sonographic findings are abnormal but inconclusive, or when patients exhibit typical symptoms despite normal imaging, further evaluation via hysteroscopy is often necessary. Hysteroscopy combined with endometrial biopsy remains the gold standard for the diagnosis of endometrial abnormalities.^[8]^

This study aims to analyze the histopathological classifications and associated influencing factors of endometrial tissue in infertile women with heterogeneous endometrial echogenicity identified on ultrasound. It also seeks to explore the clinical value of such echogenic patterns in diagnosing endometrial lesions, thereby providing evidence-based support for the clinical diagnosis and management of these patients.

## 2. Methods

### 2.1. Case source

This cross-sectional study collected survey data from 450 infertile women with heterogeneous endometrial echogenicity who attended the Department of Reproductive Medicine, First Teaching Hospital of Tianjin University of Traditional Chinese Medicine, between January 2024 and December 2024. After excluding those with incomplete information, a total of 312 cases were included in the final analysis. All patients underwent hysteroscopy and endometrial biopsy, and were categorized into the lesion group or the normal group based on histopathological findings. This study was approved by the Ethics Committee of the First Teaching Hospital of Tianjin University of Traditional Chinese Medicine (Ethics Approval No. TYLL2025[Z]字 036).

In the data analysis, Spearman’s rank correlation was first applied to assess the associations between endometrial lesions and various clinical variables. A multivariable logistic regression model was constructed to identify factors independently associated with endometrial lesions, and a separate multivariable logistic regression model was developed as the primary prediction model. A neural network was used only as a supplementary exploratory analysis of normalized predictor importance.

Restricted cubic spline (RCS) regression was used to explore potential nonlinear relationships between endometrial thickness and the occurrence of lesions. Model complexity and the ability to capture local features were assessed by comparing RCS models with different knot numbers. Locally weighted scatterplot smoothing (LOWESS) was also employed to visualize nonlinear trends.

Furthermore, forest plots were generated to illustrate associations between endometrial lesions and key variables. Predictive models were evaluated using receiver operating characteristic curves and precision-recall curves. Calibration curves and learning curves were used to assess the predictive accuracy and generalizability of the models in infertile women.

### 2.2. Diagnostic criteria

The diagnosis of infertility was based on the Guidelines for the Diagnosis of Infertility, defined as the failure to achieve a clinical pregnancy after 12 consecutive months of regular, unprotected sexual intercourse in a couple desiring conception.

The ultrasound diagnostic criteria for heterogeneous endometrial echogenicity referred to the 2015 consensus of the Morphological Uterus Sonographic Assessment group: heterogeneous endometrial echogenicity, with or without cystic structures, or homogeneous echogenicity accompanied by cystic changes. Histopathological diagnosis under microscopy included both normal endometrium and various types of abnormal endometrial lesions. Histopathological categories were not mutually exclusive because more than one pathological finding could coexist in the same patient. A patient was classified as lesion-positive if at least one abnormal histopathological finding was identified, whereas the normal group included only patients without any identified endometrial lesion. For the descriptive analysis of individual pathological findings, patients with multiple diagnoses were counted in each applicable category; therefore, the sum of category-specific counts could exceed the total number of lesion-positive patients.

### 2.3. Inclusion criteria

Participants included in this study were married women between the ages of 22 and 45 who met the diagnostic criteria for infertility and presented with heterogeneous endometrial echogenicity as defined by ultrasound standards. All patients had undergone hysteroscopic examination and endometrial tissue biopsy, and had complete and accurate clinical records available for review.

### 2.4. Exclusion criteria

Patients were excluded if they had structural abnormalities that could interfere with diagnostic interpretation, such as intrauterine adhesions or hydrosalpinx as revealed by ultrasound. Additional exclusion criteria included the presence of severe comorbidities such as cardiovascular or cerebrovascular disease, hepatic or renal insufficiency, or malignancies. Individuals who were unable to provide accurate medical histories or who declined to participate in data collection were also excluded. Moreover, patients with psychiatric disorders or cognitive impairments that interfered with questionnaire completion or clinical evaluation were not included in the final analysis.

### 2.5. TVS procedure

Transvaginal sonographic examinations were performed by a senior attending physician in the Department of Reproductive Medicine at the First Teaching Hospital of Tianjin University of Traditional Chinese Medicine, each with over 5 years of clinical experience. All ultrasounds were conducted using a Voluson S6 color Doppler ultrasound system (GE Healthcare, USA) with a probe frequency of 5 to 9 MHz. Scans were uniformly scheduled between the third and seventh day following the end of menstruation, with patients positioned in lithotomy during the procedure. The examination protocol included assessments of uterine size, endometrial morphology and thickness, the integrity of the endometrial midline, symmetry of endometrial distribution, and the presence of any abnormal echogenic patterns. The bilateral adnexal regions and pelvic cavity were also carefully evaluated.

### 2.6. Hysteroscopy examination

All patients underwent hysteroscopic examination and endometrial biopsy on the same day as the TVS, performed by the same attending physician with more than 5 years of clinical experience. Local anesthesia was administered prior to the procedure using lidocaine hydrochloride gel (I) (10 g: 0.2 g/tube; Handan Kangye Pharmaceutical Co., Ltd.), with 0.2 g applied topically to the cervix.

The uterine cavity was distended using normal saline, and intrauterine pressure was maintained below 80 mm Hg. The hysteroscopic equipment used was a continuous irrigation diagnostic system manufactured by Olympus (Japan), comprising a 4.5 mm outer sheath and a corresponding inner scope (model A4674A) with a 30° viewing angle.

During the procedure, a systematic examination was conducted from the distal to the proximal end of the uterine cavity, including assessments of the cervical canal, posterior uterine wall, fundus, bilateral uterine cornua and tubal ostia, anterior wall, and lateral walls. At the conclusion of the hysteroscopy, endometrial tissue samples were collected using a disposable intrauterine suction catheter (Jiangxi Yajie Medical Technology Co., Ltd.) and sent to the Department of Pathology at the First Teaching Hospital of Tianjin University of Traditional Chinese Medicine for routine histopathological examination and definitive diagnosis.

### 2.7. Statistical analysis

Statistical analyses of the clinical and pathological characteristics were performed using SPSS Statistics version 25.0 (IBM Corp., Armonk). Continuous variables were assessed for normality. Normally distributed continuous variables were expressed as the mean ± standard deviation and compared using the independent-samples *t* test or analysis of variance, as appropriate. Non-normally distributed continuous variables were presented as the median and interquartile range (IQR) [M (P25, P75)] and compared using the Mann–Whitney *U* test or Kruskal–Wallis test, as appropriate. Categorical variables were expressed as frequencies and percentages (n [%]) and compared using the chi-square test or Fisher’s exact test.

Spearman’s rank correlation analysis was performed to examine the associations between endometrial lesions and the included clinical variables. The outcome variable was coded as 0 for normal endometrium and 1 for the presence of an endometrial lesion. A multivariable logistic regression model was constructed to identify factors independently associated with endometrial lesions. Regression coefficients (β), adjusted odds ratios (ORs), 95% confidence intervals (CIs), and *P*-values were reported. Multicollinearity among the included predictors was evaluated using variance inflation factors.

The primary prediction model was developed using multivariable logistic regression and included 8 prespecified predictors: age, history of intrauterine procedures, number of abortions, body mass index (BMI), endometrial thickness, type of infertility, menstrual color, and menstrual blood clots. Multivariable logistic regression was selected because the study outcome was binary, the sample size was moderate, and the method generates reproducible individual predicted probabilities. In addition, its regression coefficients can be converted into ORs, facilitating interpretation of the associations between the included predictors and the presence of endometrial lesions.

Age, number of abortions, BMI, and endometrial thickness were entered into the model as continuous variables. History of intrauterine procedures, type of infertility, menstrual color, and menstrual blood clots were treated as categorical variables. No history of intrauterine procedures, primary infertility, red menstrual color, and absence of menstrual blood clots were used as the corresponding reference categories. All 8 predictors were entered simultaneously into the multivariable logistic regression model. No outcome or predictor data were missing; therefore, no missing-data imputation was performed.

The prediction model was implemented using the Logistic Regression function in scikit-learn, with the lbfgs solver and a maximum of 5000 iterations. During cross-validation, the 4 continuous predictors were standardized using the means and standard deviations calculated exclusively from each training fold, and the resulting parameters were then applied to the corresponding validation fold. Categorical predictors were dummy-encoded within each training fold using drop-first coding, and categories not observed during training were ignored in the corresponding validation fold.

Internal validation was conducted using repeated stratified 5-fold cross-validation with 20 repeats, resulting in 100 validation splits. For each split, all preprocessing and model fitting were performed using the training folds, after which predicted probabilities were generated for patients in the held-out fold. Each participant therefore contributed 20 out-of-fold predicted probabilities, and the final patient-level predicted probability was defined as the mean of these 20 probabilities. All discrimination, calibration, and classification performance measures were calculated exclusively from these patient-level out-of-fold predicted probabilities. A random seed of 20,260,725 was used to ensure reproducibility.

Model discrimination was evaluated using the area under the receiver operating characteristic curve and the area under the precision-recall curve (PR-AUC). Overall prediction error and calibration were evaluated using the Brier score, calibration intercept, calibration slope, and calibration curve. Classification performance was assessed at the conventional probability threshold of 0.50 and at the Youden-optimal threshold of 0.733 derived from the cross-validated predicted probabilities. The reported classification measures included sensitivity, specificity, positive predictive value (PPV), negative predictive value (NPV), F1-score, accuracy, and balanced accuracy. A confusion matrix was generated at the Youden-optimal threshold. Ninety-five percent CIs for the performance measures were estimated using 1000 patient-level bootstrap resamples. A learning curve was constructed to compare training and cross-validation accuracy across increasing training sample sizes.

A separate neural network model was constructed as a supplementary exploratory analysis of normalized predictor importance. The model consisted of an input layer, 2 hidden layers containing 64 and 32 neurons, respectively, and a sigmoid output layer. Rectified linear unit activation was used in both hidden layers. Model training was performed using the Adam optimizer with an initial learning rate of 0.001, a batch size of 32, and a maximum of 200 epochs. Early stopping with a patience of 20 epochs and a dropout rate of 0.30 were applied to reduce overfitting. Predictor importance was calculated using the connection-weight method and normalized to sum to 1. This neural network analysis was not used for primary model development or internal validation. The exploratory neural network included age, BMI, type of infertility, number of miscarriages, history of intrauterine procedures, menstrual cycle, menstrual color, and endometrial thickness.

RCS regression and LOWESS were used to explore the potential nonlinear association between endometrial thickness and the odds of endometrial lesions. A sensitivity analysis compared RCS models with 3 and 4 knots. Forest plots were generated to present the ORs and corresponding 95% CIs from the logistic regression analysis.

Prediction modeling and graphical analyses were conducted using Python 3.10.9. The principal packages included pandas version 2.2.3, NumPy version 2.2.5, scikit-learn version 1.7.2, statsmodels version 0.14.6, Matplotlib version 3.10.9, and SciPy version 1.15.3. All statistical tests were two-sided, and *P* < .05 was considered statistically significant.

## 3. Results

### 3.1. Endometrial pathology in infertile women with heterogeneous endometrial echogenicity

A total of 312 participants were included in the final analysis. The distribution of pathological types among patients, categorized as normal endometrium, endometritis, focal EH, hyperplasia without atypia, hyperplasia with atypia, EP, and fibroids, was as follows: normal endometrium (n = 85), endometritis (n = 175), focal EH (n = 80), hyperplasia without atypia (n = 22), hyperplasia with atypia (n = 4), polyps (n = 85), and fibroids (n = 2) (Table 1, Supplementary Figure 1, Supplemental Digital Content 1).

**Table 1:** Endometrial pathology results in patients with heterogeneous endometrial echoes.

Pathological type	Frequency (proportion/percentage)
Normal endometrium	85 (27.24%)
Endometrial polyp	85 (27.24%)
Uterine fibroid	2 (0.64%)
Endometrial hyperplasia	106 (33.97%)
Focal irregular hyperplasia	80 (25.64%)
Hyperplasia without atypia	22 (7.05%)
Hyperplasia with atypia	4 (1.28%)
Chronic endometritis	175 (56.09%)

### 3.2. Descriptive distribution of disease-related characteristics across histopathological findings

The disease-related characteristics summarized in this study included age, BMI, duration of infertility, type of infertility, number of previous miscarriages, history of intrauterine procedures, and endometrial thickness. CE was the most frequently recorded histopathological finding (n = 175), followed by EP (n = 85), focal irregular EH (n = 80), hyperplasia without atypia (n = 22), hyperplasia with atypia (n = 4), and myoma (n = 2).

The distributions of age, BMI, infertility duration, infertility type, previous miscarriages, history of intrauterine procedures, and endometrial thickness varied numerically across the histopathological categories. BMI and endometrial thickness appeared numerically higher in some polyp and hyperplasia categories than in patients with normal endometrium. However, the myoma and hyperplasia-with-atypia categories contained very small numbers of patients, and their descriptive estimates should therefore be interpreted cautiously. Because multiple histopathological findings could coexist in the same patient, the categories were not mutually exclusive and no independent between-category statistical comparisons were inferred from these descriptive data (Table 2).

**Table 2:** Baseline characteristics.

Variable	Normal endometrium		Polyp	Myoma	Endometrial hyperplasia			Chronic endometritis
					Focal irregular hyperplasia	Hyperplasia without atypia	Hyperplasia with atypia	
Cases(n)	n = 85		n = 85	n = 2	n = 80	n = 22	n = 4	n = 175
Age	32 (29~35)		32 (29~35)	34 (27~34)	32 (29.25~35)	31.5 (29.75~36.25)	38 (32.5~39.75)	32 (30~35)
BMI	22 (20~24)		24 (21.25~26)	24.9 (24.9)	23 (21~25)	24 (21.6~28)	27 (23.75~30.25)	23 (22~25)
Duration of infertility	1.794 ± 1.780		2.4 ± 2.4650	1.00 ± 0.00	2.175 ± 2.0017	2.455 ± 2.828	5.00 ± 4.6904	1 (1~3)
Types of infertility	primary	35 (11.2%)	64 (20.5%)	1 (0.3%)	51 (16.3%)	11 (3.5%)	2 (0.6%)	104 (33.3%)
	secondary	50 (16%)	21 (6.7%)	1 (0.3%)	29 (9.3%)	11 (3.5%)	2 (0.6%)	71 (22.8%)
Number of miscarriage	No history of miscarriage	39 (12.5%)	72 (23.1%)	1 (0.3%)	59 (18.9%)	14 (4.5%)	2 (0.6%)	116 (37.2%)
	Mild history of miscarriage	26 (8.4%)	11 (3.5%)	0 (0.0%)	17 (5.4%)	7 (12.2%)	1 (0.3%)	51 (16.3%)
	Moderate history of miscarriage	19 (6.1%)	2 (0.6%)	1 (0.3%)	4 (1.3%)	1 (0.3%)	1 (0.3%)	7 (2.2%)
	Severe history of miscarriage	1 (0.3%)	0 (0.0%)	0 (0.0%)	0 (0.0%)	0 (0.0%)	0 (0.0%)	1 (0.3%)
Number of intrauterine procedures	No history of intrauterine surgery	44 (14.1%)	73 (23.4%)	1 (0.3%)	61 (19.6%)	15 (4.8%)	2 (0.6%)	129 (42.3%)
	Single intrauterine surgery history	20 (6.4%)	9 (2.9%)	0 (0.0%)	15 (4.8%)	5 (1.6%)	0 (0.0%)	33 (10.6%)
	Double intrauterine surgery history	8 (2.6%)	1 (0.3%)	1 (0.3%)	2 (0.6%)	0 (0.0%)	0 (0.0%)	10 (3.2%)
	Multiple intrauterine surgery history	13 (4.2%)	2 (0.6%)	0 (0.0%)	2 (0.6%)	2 (0.6%)	2 (0.6%)	3 (1.0%)
Endometrial thickness	8.905 ± 2.9147		11 (8~13.85)	12.7 (9.6~12.7)	11.094 ± 3.6691	11.741 ± 5.2312	12.025 ± 2.4088	9.9 (7.7~12.5)

BMI = body mass index.

### 3.3. Descriptive distribution of menstrual characteristics across histopathological findings

The menstrual characteristics summarized included menstrual cycle, duration of menstruation, menstrual flow, menstrual color, menstrual blood clots, dysmenorrhea, and intermenstrual bleeding. A normal menstrual cycle, normal duration of menstruation, normal menstrual flow, and red menstrual color were the most frequently recorded patterns in most histopathological categories. Menstrual blood clots were frequently reported, whereas intermenstrual bleeding was uncommon across the categories.

Because the histopathological categories were not mutually exclusive and the same patient could contribute to more than one pathological category, these findings were interpreted descriptively. No statistically significant between-category differences were inferred because Table 3 did not include valid inferential test results. Detailed distributions of menstrual characteristics are presented in Table 3.

**Table 3:** Baseline characteristics.

Variable	Normal endometrium		Polyp	Myoma	Endometrial hyperplasia			Chronic endometritis
					Focal irregular hyperplasia	Hyperplasia without atypia	Hyperplasia with atypia	
Cases(n)	n = 85		n = 85	n = 2	n = 80	n = 22	n = 4	n = 175
Menstrual cycle	Normal	52 (16.7%)	53 (17.0%)	0 (0.0%)	53 (17.0%)	11 (3.5%)	4 (1.3%)	118 (37.8%)
	Preliminary	7 (2.2%)	6 (1.9%)	1 (0.3%)	6 (1.9%)	4 (1.3%)	0 (0.0%)	11 (3.5%)
	Late	26 (8.3%)	26 (8.3%)	1 (0.3%)	21 (6.7%)	7 (2.2%)	0 (0.0%)	46 (14.7%)
Menstrual period	Normal	67 (21.5%)	60 (19.2%)	11 (3.5%)	65 (20.8%)	9 (2.9%)	2 (0.6%)	125 (40.1%)
	Short	10 (3.2%)	14 (4.5%)	0 (0.0%)	9 (2.9%)	8 (2.6%)	0 (0.0%)	33 (10.6%)
	Long	8 (2.6%)	11 (3.5%)	22 (7.1%)	6 (1.9%)	0 (0.0%)	2 (0.6%)	17 (5.4%)
Menstrual flow	Normal	72 (23.1%)	66 (21.2%)	2 (0.6%)	64 (20.5%)	16 (5.1%)	3 (1.0%)	142 (45.5%)
	Few	8 (2.6%)	11 (3.5%)	0 (0.0%)	9 (2.9%)	5 (1.6%)	0 (0.0%)	22 (7.1%)
	Many	5 (1.6%)	8 (2.6%)	0 (0.0%)	7 (2.2%)	0 (0.0%)	1 (0.3%)	11 (3.5%)
Color of menstruation	Red	65 (20.8%)	69 (22.1%)	2 (0.6%)	66 (21.2%)	18 (5.8%)	2 (0.6%)	149 (47.8%)
	Dark	19 (6.1%)	15 (4.8%)	0 (0.0%)	13 (4.2%)	4 (1.3%)	2 (0.6%)	26 (8.3%)
Menstrual blood clots	No	21 (6.7%)	13 (4.2%)	0 (0.0%)	13 (4.2%)	7 (2.2%)	0 (0.0%)	26 (8.3%)
	Yes	64 (20.5%)	72 (23.1%)	2 (0.6%)	67 (21.5%)	15 (4.8%)	4 (1.3%)	149 (47.8%)
Dysmenorrhea	No	47 (15.1%)	42 (13.5%)	2 (0.6%)	43 (13.8%)	15 (4.8%)	3 (1.0%)	90 (28.8%)
	Yes	38 (12.2%)	43 (13.8%)	0 (0.0%)	37 (11.9%)	7 (2.2%)	1 (0.3%)	85 (27.2%)
Menstrual bleeding	No	84 (26.9%)	79 (25.3%)	2 (0.6%)	74 (23.7%)	20 (6.4%)	4 (1.3%)	165 (52.9%)
	Yes	1 (0.3%)	6 (1.9%)	0 (0.0%)	6 (1.9%)	2 (0.6%)	0 (0.0%)	10 (3.2%)

### 3.4. Exploratory neural network analysis of predictor importance for endometrial lesions in infertile patients

As a supplementary exploratory analysis, a neural network model was constructed to assess the normalized relative importance of candidate predictors for endometrial lesions. Endometrial thickness showed the highest normalized importance score (0.301), followed by BMI (0.245) and age (0.229). The normalized importance scores for menstrual cycle, history of intrauterine procedures, and number of miscarriages, menstrual color, and type of infertility were 0.059, 0.048, 0.044, 0.044, and 0.030, respectively. These results suggest that endometrial thickness, BMI, and age contributed more strongly to the neural network predictions than the other included variables. This analysis was used solely to explore the relative contributions of candidate predictors and was not used as the primary prediction model or for the main internal validation and performance evaluation, which were based on the multivariable logistic regression model (Supplementary Figure 2, Supplemental Digital Content 2).

### 3.5. Comparison of clinical and menstrual characteristics between the normal endometrium and endometrial lesion groups

A total of 312 infertile patients were included, comprising 85 patients with normal endometrium and 227 patients with at least 1 endometrial lesion. Significant between-group differences were observed in BMI, duration of infertility, number of previous abortions, history of intrauterine procedures, endometrial thickness, and type of infertility (all *P* < .05). The median BMI was higher in the endometrial lesion group than in the normal endometrium group (23.00 [22.00–25.00] kg/m^2^ vs 22.00 [20.00–24.00] kg/m^2^, *P* < .001). Endometrial thickness was also greater in the lesion group (10.40 [7.75–12.75] mm vs 8.50 [6.90–11.00] mm, *P* < .001). The distribution of infertility duration differed significantly between the groups (*P* = .028), with a higher upper quartile in the lesion group. Significant differences were also observed in the distributions of the number of previous abortions and history of intrauterine procedures (both *P* < .001). In addition, the distribution of infertility type differed between the groups (*P* = .005), with a higher proportion of primary infertility in the endometrial lesion group than in the normal endometrium group (59.0% vs 41.2%).

No statistically significant between-group differences were observed in age, menstrual cycle, duration of menstruation, menstrual flow, menstrual color, menstrual blood clots, dysmenorrhea, or intermenstrual bleeding (all *P* > .05) (Table 4). These findings represent unadjusted between-group associations and should not be interpreted as evidence of causality.

**Table 4:** Baseline clinical and menstrual characteristics of infertile patients according to endometrial pathology status.

Variable	Total (n= 312)	Normal endometrium (n = 85)	Endometrial lesion (n= 227)	*P*
Age (yr)	32.00 (29.75–35.00)	32.00 (29.00–35.00)	32.00 (30.00–35.00)	.319
BMI (kg/m^2^)	23.00 (21.00–25.00)	22.00 (20.00–24.00)	23.00 (22.00–25.00)	<.001
Duration of infertility (years)	1.00 (1.00–2.00)	1.00 (1.00–2.00)	1.00 (1.00–3.00)	.028
Number of abortions	0.00 (0.00–1.00)	1.00 (0.00–1.00)	0.00 (0.00–1.00)	<.001
History of intrauterine procedures	0.00 (0.00–1.00)	0.00 (0.00–1.00)	0.00 (0.00–1.00)	<.001
Endometrial thickness (mm)	9.60 (7.47–12.10)	8.50 (6.90–11.00)	10.40 (7.75–12.75)	<.001
Type of infertility				.005
Primary	169 (54.2)	35 (41.2)	134 (59.0)	
Secondary	143 (45.8)	50 (58.8)	93 (41.0)	
Menstrual cycle				.793
Normal	196 (62.8)	52 (61.2)	144 (63.4)	
Preliminary	116 (37.2)	33 (38.8)	83 (36.6)	
Duration of menstruation				.379
Normal	229 (73.4)	67 (78.8)	162 (71.4)	
Short	50 (16.0)	10 (11.8)	40 (17.6)	
Long	33 (10.6)	8 (9.4)	25 (11.0)	
Menstrual flow				.657
Normal	254 (81.4)	72 (84.7)	182 (80.2)	
Few	36 (11.5)	8 (9.4)	28 (12.3)	
Many	22 (7.1)	5 (5.9)	17 (7.5)	
Color of menstruation				.092
Red	258 (82.7)	65 (76.5)	193 (85.0)	
Dark	54 (17.3)	20 (23.5)	34 (15.0)	
Menstrual blood clots				.103
No	58 (18.6)	21 (24.7)	37 (16.3)	
Yes	254 (81.4)	64 (75.3)	190 (83.7)	
Dysmenorrhea				1.000
No	171 (54.8)	47 (55.3)	124 (54.6)	
Yes	141 (45.2)	38 (44.7)	103 (45.4)	
Intermenstrual bleeding				.198
No	299 (95.8)	84 (98.8)	215 (94.7)	
Yes	13 (4.2)	1 (1.2)	12 (5.3)	

### 3.6. Multivariable logistic regression analysis of risk factors for endometrial lesions in infertile patients

A multivariable logistic regression model was constructed to identify factors independently associated with endometrial lesions in infertile patients. After adjustment for age, history of intrauterine procedures, number of previous abortions, BMI, endometrial thickness, type of infertility, menstrual color, and menstrual blood clots, BMI and endometrial thickness remained independently associated with endometrial lesions.

Each 1-kg/m^2^ increase in BMI was associated with 16.2% higher odds of an endometrial lesion (β = 0.150, SE = 0.048, adjusted OR = 1.162, 95% CI: 1.056–1.277, *P* = .002). Similarly, each 1-mm increase in endometrial thickness was associated with 10.8% higher odds of an endometrial lesion (β = 0.102, SE = 0.043, adjusted OR = 1.108, 95% CI: 1.018–1.205, *P* = .017).

Age (*P* = .122), history of intrauterine procedures (*P* = .072), number of previous abortions (*P* = .188), type of infertility (secondary vs primary, *P* = .528), menstrual color (dark vs red, *P* = .332), and menstrual blood clots (yes vs no, *P* = .579) were not independently associated with endometrial lesions. Variance inflation factors for all included variables ranged from 1.025 to 3.630 and were below 5, indicating no evidence of problematic multicollinearity (Table 5).

**Table 5:** Multivariable logistic regression analysis and multicollinearity assessment of factors associated with endometrial lesions in infertile patients.

	B	S.E	*P*-value	OR (95%CI)	VIF
Age (yr)	0.057	0.037	.122	1.059 (0.985, 1.139)	1.066
History of intrauterine procedures	−0.392	0.218	.072	0.675 (0.440, 1.036)	2.342
Number of abortions	−0.476	0.362	.188	0.621 (0.306, 1.263)	3.63
BMI (kg/m^2^)	0.150	0.048	.002	1.162 (1.056, 1.277)	1.031
Endometrial thickness (mm)	0.102	0.043	.017	1.108 (1.018, 1.205)	1.062
Type of infertility (Secondary vs Primary)	0.281	0.4453	.528	1.325 (0.553, 3.171)	2.462
Color of menstruation (Dark vs Red)	−0.344	0.355	.332	0.709 (0.353, 1.422)	1.025
Menstrual blood clots (Yes vs No)	0.194	0.350	.579	1.214 (0.611, 2.412)	1.039

### 3.7. Correlation analysis of factors associated with endometrial lesions in infertile patients

Correlation analysis revealed that BMI, duration of infertility, and endometrial thickness were positively and significantly associated with the presence of endometrial lesions in infertile patients (*P* < .05). This indicates that higher BMI, longer duration of infertility, and greater endometrial thickness were associated with an increased risk of developing endometrial lesions.

In contrast, type of infertility showed a significant negative correlation with endometrial lesions (*r* = –0.158, *P* = .005), suggesting that primary infertility was inversely associated with the occurrence of endometrial lesions.

Other variables, including age, menstrual cycle, duration of menstruation, menstrual flow, menstrual color, presence of menstrual blood clots, dysmenorrhea, and intermenstrual bleeding, did not show statistically significant correlations with endometrial lesions (*P* > .05).

Overall, BMI, duration of infertility, type of infertility, and endometrial thickness emerged as important factors associated with the occurrence of endometrial lesions in infertile patients (Table 6).

**Table 6:** The correlation between endometrial lesions in infertility and included variables.

Variable	Correlation coefficient	*P*-value
Age	0.057	.318
BMI	0.214†	<.001
Duration of infertility	0.124*	.029
Types of infertility	−0.158†	.005
Endometrial thickness	0.195†	<.001
Menstrual cycle	−0.018	.752
Menstrual period	0.072	.206
Menstrual flow	0.052	.358
Color of menstruation	−0.102	.073
Menstrual blood clots	0.095	.093
Dysmenorrhea	0.008	.891
Menstrual bleeding	0.092	.105

* When the confidence level (double test) is 0.05, the correlation is significant.

† When the confidence level (double test) is 0.001, the correlation is significant.

### 3.8. Development and internal validation of the final prediction model

The final multivariable logistic regression prediction model was internally validated in 312 participants, including 227 patients with at least one endometrial lesion and 85 patients with normal endometrium. Model performance was evaluated using patient-level mean out-of-fold predicted probabilities generated from repeated stratified 5-fold cross-validation with 20 repeats.

**Figure 1.**
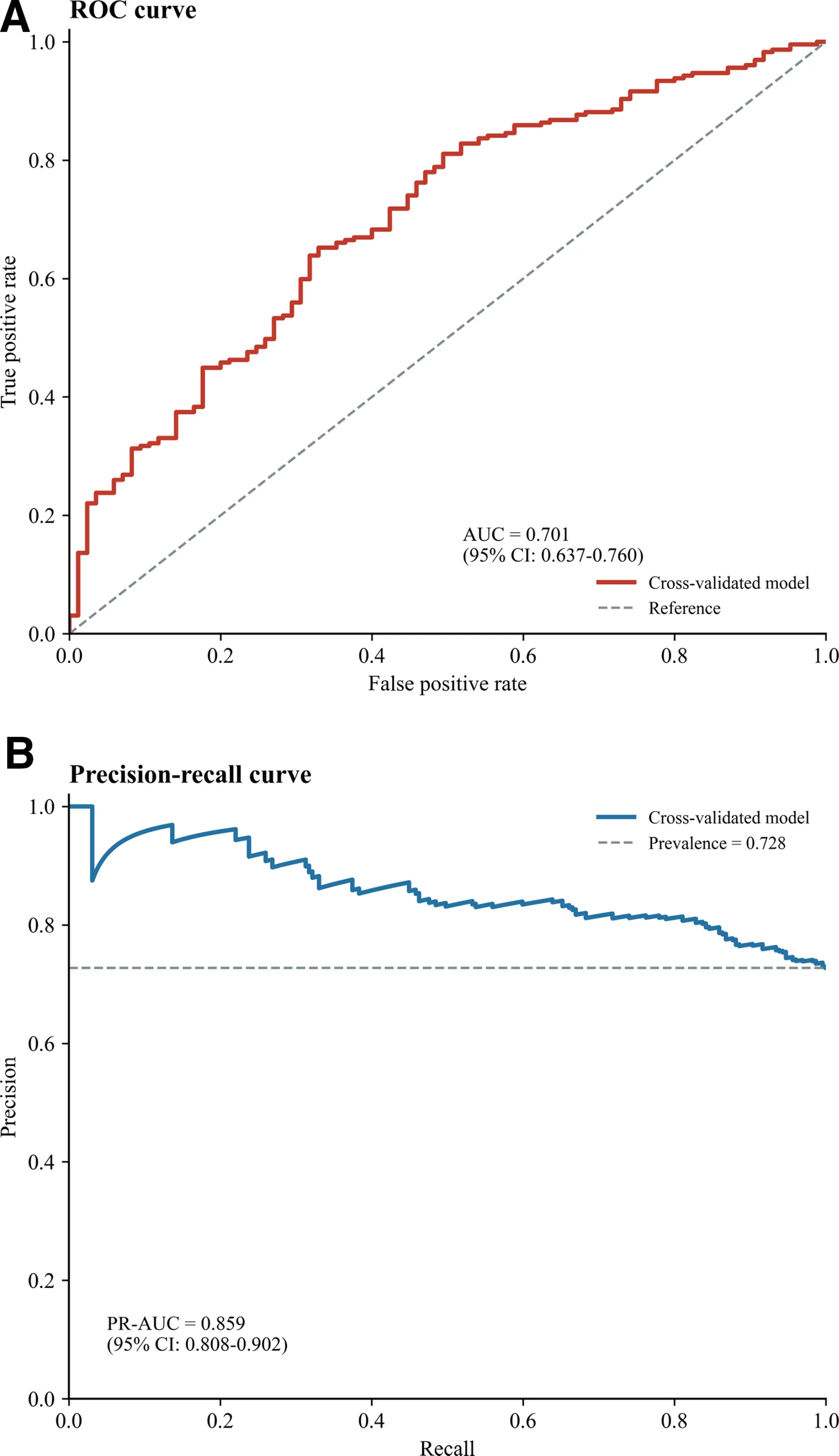
Discrimination performance of the final prediction model. (A) the area under the receiver operating characteristic curve; (B) the area under the precision-recall curve.

The model achieved a the area under the receiver operating characteristic curve of 0.701 (95% CI: 0.637–0.760), indicating moderate discrimination between patients with and without endometrial lesions. The PR-AUC was 0.859 (95% CI: 0.808–0.902), which was higher than the no-skill reference corresponding to the observed lesion prevalence of 0.728. However, the relatively high prevalence of endometrial lesions should be considered when interpreting the PR-AUC (Figs. 1A and 1B).

The Brier score was 0.180 (95% CI: 0.158–0.204), indicating the overall magnitude of prediction error. The calibration intercept was 0.168 (95% CI: −0.214–0.556), and the calibration slope was 0.810 (95% CI: 0.521–1.102). Although the CIs included the ideal calibration values of 0 and 1, respectively, the point estimates suggested slight overall underestimation of lesion probability and some overfitting. These calibration findings should therefore be interpreted cautiously.

At the conventional probability threshold of 0.50, the model showed high sensitivity of 0.920 (95% CI: 0.885–0.953) but low specificity of 0.224 (95% CI: 0.135–0.318). The PPV, NPV, F1-score, accuracy, and balanced accuracy were 0.759, 0.512, 0.832, 0.730, and 0.572, respectively. Thus, the threshold of 0.50 favored the identification of patients with endometrial lesions but had limited ability to correctly identify patients with normal endometrium.

At the Youden-optimal threshold of 0.733, sensitivity and specificity were 0.652 (95% CI: 0.590–0.716) and 0.670 (95% CI: 0.565–0.766), respectively. The PPV was 0.840, the NPV was 0.420, the F1-score was 0.734, the accuracy was 0.657, and the balanced accuracy was 0.661. Compared with the threshold of 0.50, the Youden-optimal threshold provided a more balanced trade-off between sensitivity and specificity, although the overall classification performance remained moderate. Complete internally validated discrimination, calibration, and classification performance estimates are presented in Table 7.

**Table 7:** Internally validated performance of the prediction model.

Performance metric	Estimate (95% CI)
**Discrimination and calibration**	
Sample size, n	312
ROC-AUC	0.701 (0.637–0.760)
PR-AUC	0.859 (0.808–0.902)
Brier score	0.180 (0.158–0.204)
Calibration intercept	0.168 (−0.214–0.556)
Calibration slope	0.810 (0.521–1.102)
**Classification performance at threshold = 0.50**	
Sensitivity	0.920 (0.885–0.953)
Specificity	0.224 (0.135–0.318)
PPV	0.759 (0.708–0.805)
NPV	0.512 (0.354–0.667)
F1-score	0.832 (0.797–0.863)
Accuracy	0.730 (0.683–0.776)
Balanced accuracy	0.572 (0.525–0.622)
**Classification performance at Youden-optimal threshold = 0.733**	
Sensitivity	0.652 (0.590–0.716)
Specificity	0.670 (0.565–0.766)
PPV	0.840 (0.782–0.892)
NPV	0.420 (0.339–0.500)
F1-score	0.734 (0.682–0.782)
Accuracy	0.657 (0.606–0.708)
Balanced accuracy	0.661 (0.603–0.718)

All estimates were derived from repeated stratified 5-fold cross-validation out-of-fold predicted probabilities (5 folds × 20 repeats; random seed = 20,260,725). Patient-level predicted probabilities were defined as the mean of all out-of-fold probabilities for each participant. Ninety-five percent confidence intervals were estimated using patient-level bootstrap resampling with 1000 replicates.

ROC-AUC = area under the receiver operating characteristic curve, PR-AUC = area under the precision-recall curve, PPV = positive predictive value, NPV = negative predictive value.

### 3.9. Confusion matrix analysis of the final prediction model

At the Youden-optimal probability threshold of 0.733, the confusion matrix showed that 148 of the 227 patients with endometrial lesions were correctly classified as positive, whereas 79 were incorrectly classified as negative. Among the 85 patients without endometrial lesions, 57 were correctly classified as negative and 28 were incorrectly classified as positive.

These results corresponded to an accuracy of 0.657, a PPV of 0.840, a sensitivity of 0.652, a specificity of 0.670, an F1-score of 0.734, and a balanced accuracy of 0.661. Thus, the Youden-optimal threshold provided a relatively balanced trade-off between sensitivity and specificity. Although the PPV was relatively high, the NPV was only 0.420, and the presence of 79 false-negative cases indicated that the model remained limited in ruling out endometrial lesions. The confusion matrix should therefore be interpreted together with the model’s discrimination and calibration performance (Supplementary Figure 3, Supplemental Digital Content 3 and Table 7).

### 3.10. RCS regression and LOWESS analysis of the nonlinear relationship between endometrial thickness and endometrial lesions in infertile patients

Supplementary Figure 4, Supplemental Digital Content 4 presents the nonlinear association between endometrial thickness and endometrial lesions. The red solid line represents the ORs estimated using RCS regression, with the shaded area indicating the corresponding 95% CI. The blue dashed line represents the descriptive trend obtained using LOWESS.

RCS-estimated association: The RCS curve showed that the OR for endometrial lesions increased gradually with increasing endometrial thickness and subsequently tended to plateau. At thickness values below the 10-mm reference value, the estimated OR was below 1. Above the reference value, the estimated OR increased modestly above 1. However, the confidence interval widened at the lower and upper ends of the endometrial thickness distribution, indicating greater uncertainty in these regions.

CIs: The shaded area represents the 95% CI. Around an endometrial thickness of approximately 10-mm, the CI is relatively narrow, indicating a more precise estimate of OR within this range. At both lower and higher ends of endometrial thickness, the CI widens, reflecting greater uncertainty in OR estimates.

LOWESS and RCS comparison: The LOWESS curve showed a pattern different from that of the RCS curve. The LOWESS-smoothed trend decreased across the lower-to-middle range of endometrial thickness and subsequently increased modestly at higher thickness values, whereas the RCS-estimated OR increased gradually and then tended to plateau. These differences may reflect the greater local flexibility of LOWESS and the smoother, model-based functional form imposed by RCS. Therefore, the 2 curves should be interpreted as complementary descriptive analyses rather than as evidence of closely concordant trends (Supplementary Figure 4, Supplemental Digital Content 4).

Supplementary Figure 5, Supplemental Digital Content 5 compares the OR–endometrial thickness curves generated by RCS models with 3 knots (red) and 4 knots (blue).

Curve shape differences: The 3-knot model produces a relatively smooth curve with a gradual trend, whereas the 4-knot model shows more pronounced undulations, capturing additional localized variations. For instance, around an endometrial thickness of approximately 7.5 mm, the 4-knot curve exhibits a marked dip that is absent in the 3-knot curve, highlighting its greater sensitivity to localized changes (Supplementary Figure 5, Supplemental Digital Content 5).

### 3.11. Calibration curve analysis of the final prediction model

Supplementary Figure 6, Supplemental Digital Content 6 presents the calibration curve based on the cross-validated predicted probabilities of the final multivariable logistic regression model. The dashed diagonal line represents perfect agreement between predicted probabilities and observed proportions, the solid green curve represents the cross-validated model, and the shaded area represents the bootstrap 95% confidence interval.

The calibration intercept was 0.168 and the calibration slope was 0.810, compared with the ideal values of 0 and 1, respectively. The positive calibration intercept suggests slight overall underestimation of the probability of endometrial lesions, whereas the calibration slope below 1 indicates that the predicted probabilities were somewhat too extreme, suggesting mild model overfitting. The calibration curve fluctuated around the ideal line across the evaluated probability range, with both underestimation and overestimation observed in different probability intervals. The relatively wide bootstrap CIs, particularly in some intermediate- and high-probability regions, indicate uncertainty in the calibration estimates. Overall, the model showed moderate but imperfect calibration, and its predicted probabilities should be interpreted cautiously (Supplementary Figure 6, Supplemental Digital Content 6).

### 3.12. Distribution differences of factors associated with endometrial lesions in infertile patients

Supplementary Figure 7, Supplemental Digital Content 7 presents boxplots comparing the distributions of BMI, endometrial thickness, and age between 2 pathological diagnostic categories: category 0 (normal endometrium, blue) and category 1 (endometrial lesions, orange).

BMI distribution: In category 0, the median BMI was approximately 22.5, with a relatively narrow IQR and limits of about 18 (lower) and 30 (upper). In category 1, the median BMI was approximately 25, with a slightly larger IQR compared with category 0, and limits of about 20 (lower) and 35 (upper).

Endometrial thickness distribution: In category 0, the median endometrial thickness was around 10-mm, with a relatively small IQR, ranging from approximately 6 to 15 mm. In category 1, the median was about 12 mm, with a slightly larger IQR, ranging from approximately 8 to 20 mm.

Age distribution: In category 0, the median age was approximately 32 years, with a relatively small IQR and limits of about 25 (lower) and 40 (upper). In category 1, the median age was approximately 34 years, with a slightly larger IQR, ranging from about 26 to 42 years.

Overall, the medians and upper limits for BMI, endometrial thickness, and age were slightly higher in patients with endometrial lesions compared with those with normal endometrium, suggesting that higher values in these parameters are more common among patients with lesions (Supplementary Figure 7, Supplemental Digital Content 7).

### 3.13. Learning curve analysis of the final prediction model

Supplementary Figure 8, Supplemental Digital Content 8 presents the learning curves of the final prediction model across different training sample sizes. The horizontal axis represents the number of training samples, and the vertical axis represents classification accuracy. At the smallest training sample size, the training accuracy was approximately 0.77, whereas the cross-validation accuracy was approximately 0.74, indicating a modest initial generalization gap.

As the training sample size increased, the training accuracy decreased and subsequently stabilized at approximately 0.74 to 0.75. The cross-validation accuracy fluctuated within a relatively narrow range of approximately 0.73 to 0.74 and did not show a sustained increase with additional training samples. The gap between the training and cross-validation curves narrowed as the training sample size increased and was approximately 0.02 at the largest sample size. The substantial overlap between the uncertainty bands of the 2 curves also suggests limited overfitting.

Overall, the learning curves indicate relatively stable but moderate generalization performance. The apparent performance plateau suggests that substantial improvement may require additional informative predictors or refinement of the model specification rather than simply increasing the sample size within the range evaluated. These findings should be interpreted together with the discrimination, calibration, and confusion matrix results (Supplementary Figure 8, Supplemental Digital Content 8).

## 4. Discussion

Uterine factors play a crucial role not only in embryo implantation but also in early pregnancy loss. Studies have shown that approximately 2-thirds of implantation failures are attributable to reduced endometrial receptivity,^[9]^ and uterine abnormalities account for 15% to 20% of cases in patients with recurrent pregnancy loss.^[10]^ Therefore, accurate identification of endometrial lesion types can provide valuable guidance for decision-making in assisted reproductive therapies. TVS is one of the most widely used noninvasive tools for evaluating endometrial structure and function in clinical practice. Parameters such as endometrial thickness, morphology, classification, peristalsis, and sub-endometrial blood flow are all critical indicators of receptivity. Among them, heterogeneous endometrial echogenicity often suggests underlying pathology;^[7,11]^ however, specific pathological correlates of this sonographic feature remain underreported in the literature.

In healthy women, the endometrium undergoes a complex cyclic process of proliferation, secretion, and regeneration, regulated by hormonal, immunologic, and metabolic factors. Heterogeneous echogenicity may be observed not only during the secretory phase of a normal menstrual cycle but also in cases of abnormal EH.^[12]^ Due to differences in study populations, pathological findings associated with heterogeneous endometrial echogenicity may vary; however, benign lesions remain the most frequently identified conditions.^[13,14]^ In the present study, CE was found to be the most frequent pathological type among patients with heterogeneous echogenicity. CE is a chronic inflammatory condition characterized by plasma cell infiltration and has been shown to significantly increase the risk of infertility, recurrent implantation failure, and recurrent pregnancy loss.^[15,16]^ The underlying mechanisms may involve disruption of the local immune microenvironment, activation of inflammatory pathways, and impaired decidualization.^[17]^

Previous studies have reported frequent co-occurrence of CE, EP, and EH and have proposed inflammatory, immune, and hormonal mechanisms that may link these conditions.^[18–20]^ In the present study, these pathological findings could coexist in the same patient. However, because the categories overlapped and the study was cross-sectional, our data cannot determine the temporal sequence of these lesions or establish that 1 pathological condition caused another.

The exploratory neural network analysis identified endometrial thickness as the predictor with the highest normalized importance score (0.301). Multivariable logistic regression showed that greater endometrial thickness was independently associated with higher odds of endometrial lesions (adjusted OR = 1.108, 95% CI: 1.108–1.205, *P* = .017). Given the cross-sectional design, this association should not be interpreted as evidence that increased endometrial thickness causes endometrial lesions. These findings align with previous research. For example, a study by Zhang G et al reported that higher endometrial thickness was associated with an increased risk of EH, and when the thickness exceeded 15 mm, its PPV markedly improved.^[21]^ Moreover, several studies have confirmed that endometrial thickness is an independent risk factor for endometrial abnormalities in women with abnormal uterine bleeding.^[22,23]^ Our RCS regression analysis further revealed a nonlinear relationship between endometrial thickness and lesion risk, with the estimated OR increasing modestly above the 10-mm reference value, although uncertainty increased at the distributional extremes. This trend may be related to underlying hormonal dysregulation or changes in the endometrial microenvironment.

Higher BMI was also independently associated with greater odds of endometrial lesions in the present study (adjusted OR = 1.162, 95% CI: 1.056–1.277, *P* = .002). Obesity is associated with altered estrogen metabolism, leading to persistently elevated levels of biologically active estrogen, which in turn promotes continuous endometrial proliferation. Retrospective cohort studies have shown that women with a BMI ≥30 kg/m^2^ are approximately 4 times more likely to develop endometrial pathology than women of normal weight,^[24]^ and weight-loss interventions have been shown to reverse endometrial abnormalities in some cases.^[25]^ Additionally, obesity frequently coexists with metabolic syndrome, another key contributor to endometrial dysregulation. Evidence suggests that increasing degrees of insulin resistance are significantly associated with a heightened risk of endometrial lesions. In vivo studies have demonstrated that metabolic syndrome disrupts the estrous cycle, increases progesterone levels, and induces EH, all of which impair reproductive success. Notably, metformin therapy reverses these effects, highlighting the impact of metabolic dysfunction on endometrial architecture and function.^[26]^ Moreover, insulin resistance and its associated metabolic abnormalities, including hyperlipidemia, have been shown to promote abnormal endometrial proliferation. Li et al (2023) reported that dysregulated lipid metabolism plays a pivotal role in the pathogenesis of endometrial carcinoma, acting through enhanced estrogen production and the activation of oncogenic signaling pathways.^[27]^ Furthermore, comprehensive analysis by Zhang et al (2021) revealed that metabolic factors such as elevated BMI, hyperuricemia, hyperlipidemia, and insulin resistance are significant risk factors for atypical EH and endometrial cancer. Their predictive model demonstrated strong discriminatory capacity, further emphasizing the importance of metabolic control in reducing endometrial pathology risk.^[28]^

A history of intrauterine procedures and the number of previous miscarriages were associated with the presence of endometrial lesions in the unadjusted analyses (both *P* < .001). However, because this study employed a cross-sectional design, these associations do not establish a temporal or causal relationship. Residual confounding and reverse causation cannot be excluded. Previous studies have suggested that repeated intrauterine procedures may be associated with endometrial injury, scarring, or infection, which may in turn be related to CE and impaired endometrial function.^[29,30]^ CE has also been frequently reported among patients with recurrent implantation failure or recurrent pregnancy loss.^[31–35]^Nevertheless, these potential mechanisms were not directly evaluated in the present study, and the absence of follow-up data prevented us from assessing subsequent implantation, pregnancy, miscarriage, or live birth outcomes.^[36]^

This study also identified age as an important predictor of endometrial pathology. With advancing age, ovarian function declines, leading to reductions in both the quantity and quality of oocytes, as well as hormonal fluctuations that affect endometrial receptivity. Studies have shown that older women tend to have abnormally elevated serum estradiol levels during the follicular phase, but reduced estradiol and progesterone levels during the secretory phase.^[37]^ These hormonal changes are often accompanied by downregulation of endometrial estrogen and progesterone receptors,^[38]^ along with upregulation of pro-inflammatory cytokines,^[39]^ ultimately contributing to impaired receptivity. Epidemiological studies have also revealed that the incidence of endometrial-related disorders increases with age, with the risks of EP and EH closely linked to advanced maternal age.^[40–42]^

The findings of this study offer valuable guidance for the individualized management of infertility patients. First, promoting childbearing at an appropriate reproductive age is essential. While age-related ovarian decline and oocyte aging are major contributors to failed pregnancies in older women, impaired endometrial receptivity has been identified as a key limiting factor for successful conception in this population.^[43]^ Encouraging timely family planning not only reduces maternal and neonatal health risks but also supports broader strategies aimed at mitigating the challenges of population aging and ensuring sustainable social development.

Second, routine monitoring of endometrial thickness is recommended. Clinicians should pay close attention to dynamic changes in endometrial thickness and employ both ultrasound and hysteroscopy to identify abnormalities early. Timely intervention in patients with extreme endometrial thickness (e.g., <7 mm or >14 mm) may improve reproductive outcomes.^[44,45]^

Third, weight management should be emphasized. For patients with elevated BMI, lifestyle modification or pharmacological interventions aimed at improving metabolic health may indirectly enhance the endometrial microenvironment.^[46]^ Lifestyle intervention has already been established as a first-line strategy in numerous clinical guidelines for infertility management.^[47,48]^

## 5. Limitations

This study has several limitations. First, it was a single-center cross-sectional study that included only infertile women with heterogeneous endometrial echogenicity who underwent hysteroscopy and biopsy. This recruitment process may have introduced selection and referral bias and may limit the generalizability of the findings to other infertility populations or clinical settings. Second, the cross-sectional design precluded assessment of temporal relationships; therefore, the observed associations cannot be interpreted as causal. Third, several pathological findings could coexist in the same patient, and some rare subtypes, including atypical hyperplasia and fibroids, were represented by only a small number of cases, limiting the precision and reliability of category-specific comparisons. Fourth, although the prediction model underwent repeated cross-validation for internal validation, it was not externally validated in an independent population, and its transportability remains uncertain. Finally, follow-up data on reproductive outcomes, including implantation, clinical pregnancy, miscarriage, and live birth, were unavailable. Consequently, the prognostic implications of the identified lesions and whether their treatment improves reproductive outcomes could not be evaluated. Future multicenter prospective studies should externally validate the prediction model and incorporate longitudinal reproductive outcomes.

## Author Contributions

**Conceptualization:** Saihua Ma.

**Formal analysis:** Saihua Ma, Jiao Liu, Zheng Gong.

**Investigation:** Rong Dong, Baojuan Wang.

**Data curation:** Jiao Liu, Jingyu Xu, Zheng Gong.

**Methodology:** Jingyu Xu.

**Supervision:** Linlin Fan.

**Writing – original draft:** Saihua Ma.

**Writing – review & editing:** Saihua Ma, Tian Xia.
















